# Fractal Feature of Particle-Size Distribution in the Rhizospheres and Bulk Soils during Natural Recovery on the Loess Plateau, China

**DOI:** 10.1371/journal.pone.0138057

**Published:** 2015-09-14

**Authors:** Zilin Song, Chao Zhang, Guobin Liu, Dong Qu, Sha Xue

**Affiliations:** 1 College of Natural Resources and Environment, Northwest A&F University, Yangling, China; 2 State Key Laboratory of Soil Erosion and Dryland Farming on the Loess Plateau, Institute of Soil and Water Conservation, Northwest A&F University, Yangling, China; Leibniz-Institute of Vegetable and Ornamental Crops, GERMANY

## Abstract

The application of fractal geometry to describe soil structure is an increasingly useful tool for better understanding the performance of soil systems. Only a few studies, however, have focused on the structure of rhizospheric zones, where energy flow and nutrient recycling most frequently occur. We used fractal dimensions to investigate the characteristics of particle-size distribution (PSD) in the rhizospheres and bulk soils of six croplands abandoned for 1, 5, 10, 15, 20, and 30 years on the Loess Plateau of China and evaluated the changes over successional time. The PSDs of the rhizospheres and the fractal dimensions between rhizosphere soil and bulk soils during the natural succession differed significantly due to the influence of plant roots. The rhizospheres had higher sand (0.05–1.00 mm) contents, lower silt (<0.002 mm) contents, and lower fractal dimensions than the bulk soils during the early and intermediate successional stages (1–15 years). The fractal dimensions of the rhizosphere soil and bulk soil ranged from 2.102 to 2.441 and from 2.214 to 2.459, respectively, during the 30-year restoration. Rhizospheric clay and silt contents and fractal dimension tended to be higher and sand content tended to be lower as abandonment age increased, but the bulk soils had the opposite trend. Linear regression analysis indicated that the fractal dimensions of both the rhizospheres and bulk soils were significantly linearly correlated with clay, sand, organic-carbon, and total-nitrogen contents, with *R* ^2^ ranging from 0.526 to 0.752 (*P*<0.001). In conclusion, PSD differed significantly between the rhizosphere soil and bulk soil. The fractal dimension was a sensitive and useful index for quantifying changes in the properties of the different soil zones. This study will greatly aid the application of the fractal method for describing soil structure and nutrient status and the understanding of the performance of rhizospheric zones during ecological restoration.

## Introduction

Particle-size distribution (PSD) of soil is closely associated with soil structure and function. Smaller soil particles are more prone to wind erosion. The loss of soil by wind erosion leads to the loss of immeasurable ecological services along with the soil fractions. Wind erosion transports soil particles and nutrients and reduces ecosystemic functioning, further decreasing the water-holding capacity of the soil, destroying soil structure, and depleting biological attributes [1–3].Therefore, PSD is an important physical parameter of soils. The ability to quantitatively describe soil PSD is important for research into soil structure, and changes in PSD can help to determine the extent to which soils are disturbed. The evaluation of soil structure and function by a single parameter is a question of great interest when monitoring soil changes. Individual parameters (e.g. clay and fine fractions), textual analysis, and soil nutrients (organic C and total N) have been widely used to characterize soil quality [2, 4]. Among these methods, the individual parameters were usually clay and silt particles and not coarse particles, which could lead to an information gap about the soil texture. Organic C is an important soil property in the assessment of soil quality but is insensitive to environmental variation on short timescales. Traditional methods thus cannot quantitatively characterize the fundamental attributes using a single parameter [5].

The possibility of characterizing soil PSD using fractal theory has recently been explored, and this method has become a useful approach for quantifying soil structure. Fractal theory provides a better understanding of the performance of a soil system, such as erodibility, water permeability, and porosity [6–11]. Recent studies have shown that the fractal dimensions of soil PSD correlated well with other soil properties. For example, Xu et al. [12] reported a strong positive correlation between the fractal dimension (*D*) and the silt and clay contents, with increasing *D* corresponding to higher silt and clay contents. Gao et al. [2] found that *D* was significantly linearly correlated with bulk density, total porosity, and organic-carbon (C) content and was a sensitive and useful index for quantifying changes in soil properties that also implied desertification. Peng et al. [13] investigated soil PSDs under different land-use patterns in wetlands of the Yellow River Delta in China using the fractal parameter and found that *D* was directly proportional to clay content, inversely proportional to sand content, and significantly correlated with organic-matter content.

Soil structure has been well studied with fractal theory, but only a few studies have applied *D* to soil PSDs during ecological restoration, especially in the rhizospheric zones where energy flow and nutrient recycling most frequently occur. The rhizosphere is the region of soil adhering closely to plant roots [14]. The properties of the soil in this region differ from those of the bulk soil due to root penetration and exudates. Rhizospheric performance is important for the functioning of ecosystems, in both direct interactions with plants (both beneficial and deleterious to growth) and in the cycling of nutrients and organic matter [15–17]. The rhizosphere has thus become the focus in field studies of soils and plants.

The Loess Plateau of China, an ecologically fragile area, has suffered from severe soil erosion. The erosion led to the loss of most of the topsoil, which reduced vegetation cover and exposed parental materials or soils with low nutrient content. The Chinese government realized the seriousness of this problem and initiated the nationwide “Grain for Green” project in 1999 to control the erosion and restore the ecological environment. Many croplands with slopes >15° were abandoned to return to green land by natural succession without anthropogenic intervention. Extensive research has recently evaluated the effectiveness of this project and has mostly indicated that this conversion has contributed greatly to the improvement of soil conditions and to the effective protection against erosion [18]. These studies, however, have focused on the properties of bulk soil, such as aggregate structure [19], water-holding capacity [20, 21], nutrient status [22, 23], and biological properties [24], while available information on the properties of rhizospheres is scarce. Such information is important for the understanding of the essence of natural succession and for the appropriate management and conservation of the ecological environment.

We characterized the PSDs in the rhizospheres using the fractal dimension during the natural recovery of six sloped croplands in a successional gradient on the Loess Plateau abandoned for 1–30 years. Our objectives were to (i) compare the characteristics of PSD and fractal dimensions between rhizosphere soil and bulk soil during natural recovery, and (ii) evaluate the changes in PSDs and fractal dimensions of rhizospheres and bulk soils over successional time and determine their relationships with selected soil properties.

## Materials and Methods

### Ethics statement

This study was conducted at the Dunshan watershed on the Loess Plateau (109°19′23′′E, 36°51′30′′N) of China. The Dunshan watershed belongs to the Ansai Research Station of Soil and Water Conservation, Northwest A&F University. The authors have been working in the Northwest A&F University, and we got permission for the filed research from the university. No specific permissions were required for these locations because our study was the investigation of vegetation and soil sampling. Our study did not involve endangered or protected species.

### Description of study area

The climate in the study area is temperate semiarid with heavy seasonal rain and periodic drought. Average annual rainfall at the study site is 510 mm, more than 60% of which falls from July to September. The mean annual temperature is 8.8°C, and the annual evaporation ranges from 1500 to 1800 mm. The topography, soil type, and soil and land-use patterns of the watershed are typical of the Loess Plateau. The soil type is Huangmian soil (Calcaric Cambisols, FAO) originating from wind-blown deposits and characterized by a yellow color, an absence of silty texture, looseness, macroporosity, and wetness-induced collapsibility. These characteristics have contributed greatly to the severe soil erosion. Typical vegetation includes herbs such as *Stipa bungeana* and *Artemisia sacrorum*, shrubs such as *Caragana korshinskii* and *Hippophae rhamnoides*, and woody plants such as *Robinia pseudoacacia* and *Caragana microphylla*.

### Experimental design and soil sampling

The substitution of space for time is an effective way for studying the changes in soil conditions and plant communities in similar soils under similar climatic conditions along a vegetative chronosequence [25] and has been widely applied in ecosystemic research [26–28]. We used this approach based on a well-dated chronosequence of abandoned croplands to investigate the PSD characteristics of rhizospheres over time. In September 2008, six croplands abandoned for 1, 5, 10, 15, 20, and 30 years were selected as the experimental sites. The lengths of abandonment were verified by local villagers and related land documents. The sites had similar topographic characteristics (gradient elevations and slopes) and had been subjected to similar farming practices before abandonment. All investigated soils had developed from the same parental materials. The croplands had been farmed for >40 years. After abandonment, the croplands were not interfered by human activities but were allowed to undergo natural vegetation succession. We have previously investigated the dynamics of the vegetation communities of these abandoned croplands over time and found that in the 1-year abandoned cropland, the vegetation community was dominated by *A*. *capillaries*, while as the *H*. *altaicus* emerged into community, vegetation community was dominated by *A*.*capillaries* and *H*. *altaicus* in the 5- and 10- year sites. *A*. *sacrorum* and *S*. *bungeana* were the important species in the middle-late stage and they coexisted in the 15- and 20-year sites, however, in the 30-year site, *A*. *sacrocrum* completely replaced *S*. *bungeana* to be the dominant species. In general, the vegetation transformed from communities dominated by *A*. *capillaries* to communities dominated by *A*. *sacrorum* over 30 years of restoration [26]. Based on this successional sequence, the present study investigated the PSDs of the rhizospheres of the two dominant species during the natural recovery. Vegetation characteristics and basic soil chemical properties are shown in Tables 1 and 2.

**Table 1:** Change of plant characteristics during the natural succession [26].

Abandoned cropland	Dominant species / Main companion species	Coverage (%)	Aboveground biomass (g m^-2^)	Root biomass (g m^-2^)
1- yr	*A*. *capillaries*	75.2 ± 10.4	454.5 ± 27.9	181.9 ± 26.0
5- yr	*A*.*capillaries H*. *altaicus*	37.4 ± 3.0 12.6 ±1.8	128.3 ± 17.9 60.5 ± 8.4	67.4 ± 10.0 20.1 ± 3.4
10- yr	*H*.*altaicus A*. *capillaries*	31.7 ±4.1 22.5 ± 2.8	131.5 ± 12.5 100.8 ± 14.1	48.6 ± 4.7 36.3 ± 5.9
15- yr	*A*.*sacrorum S*. *bungeana*	30.4 ± 3.8 3.6 ± 1.2	235.5 ± 13.7 22.5 ± 3.01	183.8 ± 21.2 20.4 ± 3.1
20- yr	*S*.*bungeana A*. *sacrorum*	30.8 ± 4.7 10.2 ± 1.6	256.8 ± 29.7 68.4 ± 6.9	81.5 ± 9.6 59.7 ± 6.3
30- yr	*A*. *sacrorum*	68.6 ± 5.6	270.2 ± 20.5	199.8 ± 17.1

The results was expressed as means ± standard deviations (n = 3). *A*. *capillaries*: *Artemisia capillaries; A*. *sacrorum*: *Artemisia sacrorum; H*. *altaicus*: *Heteropappus altaicus; S*. *Bungeana*: *Stipa bungeana*

**Table 2:** Change of soil organic C and total N during the natural succession [26].

Abandoned cropland	Dominant species/Main companion species	Soils	Organic C (g kg^-1^)	Total N (g kg^-1^)
1-yr	*A*. *capillaries*	Rhizophere	3.44 ± 0.25	0.44 ± 0.06
		Bulk	2.99 ± 0.32	0.37 ± 0.02
5-yr	*A*. *capillaries*	Rhizophere	4.63 ± 0.44	0.52 ± 0.02
	*H*. *altaicus*	Rhizophere	4.14 ± 0.40	0.45 ± 0.02
		Bulk	3.29 ± 0.62	0.36 ± 0.01
10-yr	*H*. *altaicus*	Rhizophere	4.85 ± 0.50	0.55 ± 0.05
	*A*. *capillaries*	Rhizophere	4.13 ± 0.31	0.48 ± 0.05
		Bulk	3.16 ± 0.34	0.39 ± 0.01
15-yr	*A*. *sacrorum*	Rhizophere	5.88 ± 0.21	0.64 ± 0.08
	*S*. *bungeana*	Rhizophere	4.96 ± 0.41	0.57 ± 0.02
		Bulk	4.40 ± 0.43	0.48 ± 0.07
20-yr	*S*. *bungeana*	Rhizophere	8.80 ± 0.61	0.78 ± 0.02
	*A*. *sacrorum*	Rhizophere	6.64 ± 0.52	0.70 ± 0.05
		Bulk	5.65 ± 0.55	0.65 ± 0.07
30-yr	*A*. *sacrorum*	Rhizophere	7.87 ± 0.35	0.80 ± 0.09
		Bulk	5.36 ± 0.68	0.61 ± 0.05

The results was expressed as means ± standard deviations (n = 3).

Three 20 m × 20 m plots were established at each site and were considered three replicates, given that the distance between them exceeded the spatial dependence of most soil chemical and microbial variables [29, 30]. Five 1 m× 1 m quadrants were randomly selected in each plot for the measurement of the vegetation characteristics. In each quadrant, the coverage, aboveground biomass (dry-weight), and root biomass (dry-weight) were separately recorded according to species. Rhizosphere soil of plant was extracted by pulling the plant out from the ground and separating the soil, closely adhering to the roots, with a pair of tweezers. Six individual plants were taken from each plot and the rhizosphere soil of each plant was mixed into one for the laboratory analysis. Bulk soils were collected from the top 20 cm of the soil profile apart approximately 15 cm from the plants (avoiding visible roots). The soil samples were air-dried and passed through both a 0.25- and a 1.0-mm sieve after removing the roots, stones, and debris.

### Laboratory analysis

The soil organic-C and total-nitrogen (N) contents were measured as described by Zhang et al. [31]. Soil-particle composition was measured by a laser particle-size analyzer (Mastersizer 2000, Malvern Instruments, Malvern, England). Based on the classification of soil-size fractions, the soil PSD was described according to the percentages of clay (<0.002 mm), fine silt (0.002–0.2 mm), coarse silt (0.02–0.05 mm), very fine sand (0.05–0.25 mm), fine sand (0.25–0.5 mm), medium-grained sand (0.5–1 mm), and coarse sand (1–2 mm).

### Soil fractal theory

The fractal dimension can be defined by the relationship between number and size in a statistically self-similar system [32, 33]:

$$N(X>x_{i})=kx_{i}{}^{-\text{D}}$$ (1)

where *N* (*X*≥*x*_*i*_) is the cumulative number of objects or fragments larger than size *x*_*i*_, *k* is the number of elements at a unit length scale, and *D* is the fractal dimension. The applicability of Eq (1) for PSD analysis, however, is limited due to the incomplete and inaccurate calculations of N from conventional experimental data. To solve this problem, Tyler and Wheatcraft [34] determined *D* by the Eq (2):

$$M(r<R_{i})/M_{T}={\left(R_{i}/R_{\max}\right)}_{m}{}^{3-\text{D}}$$ (2)

where M is the cumulative mass of particles of size class *i* for *r* less than *R*_*i*_, *R*_*i*_ is the average particle diameter (mm) of size class *i*, *M*_*T*_ is the total mass, and *R*_max_ is the mean diameter of the largest particle. The logarithms of both sides in Eq (2) produce the Eq (3) and the *D* were determined [7]:

$$D=3-\frac{\log M(r<R_{i})/M_{T}}{\log(R_{i}/R_{\max})}$$ (3)

### Statistical analysis

Differences between means were evaluated by one-way analyses of variance. Comparisons among means used Duncan’s multiple-range tests, calculated at *P*<0.05. Simple linear regression was performed to identify the relationships between *D* and the selected soil properties. Pearson correlation coefficient and 2-tailed test were used to distinguish the correlation and significant differences between PSDs and plant characteristics. All analyses were conducted with SPSS 17.0 (SPSS Inc., Chicago, USA).

## Results

### Comparison of PSD and *D* between rhizosphere and bulk soil

Table 3 presents the PSDs and *D*s between the rhizospheres and bulk soils for the sites with different abandonment ages. In the 1- and 30-year sites, the plant community was dominated by a single species (Table 1); the coverage of the other species was too small to take into consideration. Thus, in these two sites, only the rhizosphere and bulk soil of the dominant species were investigated. Silt was clearly the dominant soil particle in both rhizosphere soils of dominant and companion species (52.1–55.9% of the total soil mass) and bulk soil (54.6–60.7%). The silt (0.002–0.05 mm) contents were significantly lower (*P*<0.05) in the two rhizosphere soils than the bulk soil at the 1–20-year sites but were not significantly different among the three soils at the 30-year site. By contrast, the sand (0.05–1 mm) contents were significantly higher in the two rhizosphere soils than the bulk soil at the 1–15-year sites but not at the 20- and 30-year sites. Clay (<0.002 mm) contents differed significantly among the three soils at the 1-, 15-, and 20-year sites and were higher in the bulk soils at the 1- and 15-year sites. *D* for the rhizospheric and bulk-soil particles at the various abandoned sites ranged from 2.102 to 2.441 and from 2.214 to 2.459, respectively. All coefficients of determination (*R* ^2^) for the regression equations were >0.850. The bulk soils had significantly higher *D*s than the two rhizospheres at the 1–15-year sites but not at the 20- and 30-year sites.

**Table 3:** Particle size distribution (PSD) and fractal dimension (*D*) of rhizosphere soil and bulk soil during the natural succession.

Sites	Dominant species/ Companion species	Soils	Clay % (<0.002 mm)	Silt % (0.002–0.05mm)	Sand % (0.05–1mm)	Fractal dimension (*D*)	*R* ^*2*^
1-yr	*A*.*capillaries*	Rhizosphere	10.03±1.26 b	52.85±1.02 b	37.12±3.12 a	2.102±0.025 b	0.916
		Bulk	16.39±1.13 a	60.76±2.41 a	22.85±2.07 b	2.459±0.021 a	0.906
5-yr	*A*.*capillaries*	Rhizosphere	11.83±1.87 a	54.12±0.99 b	34.05±2.26 a	2.119±0.011 b	0.946
	*H*. *altaicus*	Rhizosphere	12.17±1.15 a	53.23±1.24 b	34.60±2.54 a	2.224±0.024 b	0.910
		Bulk	14.09±1.04 a	60.07±1.16 a	25.21±1.65 b	2.451±0.055 a	0.898
10-yr	*H*. *altaicus*	Rhizosphere	12.25±1.18 a	53.02±1.20 b	35.73±1.49 a	2.319±0.022 b	0.906
	*A*.*capillaries*	Rhizosphere	12.76±1.05 a	55.06±0.97 b	32.18±1.44 b	2.336±0.017 b	0.912
		Bulk	13.27±1.23 a	58.58±0.57 a	28.14±1.42 c	2.394±0.024 a	0.933
15-yr	*A*. *sacrorum*	Rhizosphere	9.41±2.00 b	54.84±1.02 b	35.75±1.13 a	2.307±0.017 b	0.885
	*S*. *bungeana*	Rhizosphere	8.77±1.88 b	54.10±1.15 b	37.13±2.06 a	2.340±0.015 b	0.899
		Bulk	13.08±1.11 a	58.51±1.66 a	28.41±0.77 b	2.362±0.029 a	0.903
20-yr	*S*. *bungeana*	Rhizosphere	16.62±1.24a	52.11±0.99 b	31.17±2.25 a	2.410±0.072 a	0.923
	*A*. *sacrorum*	Rhizosphere	15.59±1.62 a	52.64±1.37 b	31.77±2.01 a	2.418±0.064 a	0.911
		Bulk	11.37±0.53 b	55.98±1.16 a	32.65±1.47 a	2.337±0.089 a	0.900
30-yr	*A*. *sacrorum*	Rhizosphere	14.78±1.44 a	55.87±1.03 a	29.35±3.12 a	2.441±0.057 a	0.916
		Bulk	11.11±2.72 a	54.63±1.47 a	34.26±2.32 a	2.214±0.109 a	0.921

The results was expressed as means ± standard deviations (n = 3). Different letters indicate the significant difference between the rhizosphere soil and bulk soil at *p*<0.05. *R* ^2^ is the coefficient of determination.

### Change of soil PSD and *D* over successional time

Clearly, the vegetation transformed from communities dominated by *A*. *capillaries* to communities dominated by *A*. *sacrorum* over 30 years of restoration (Table 1). The pioneer species, *A*. *capillaries*, was an important dominant species from the 1st to the 15th year, and its coverage, biomasses of aboveground and roots were significantly declined across the time (*F* = 6.26, *P*<0.01). The middle-late species, *A*. *sacrorum*, was an important dominant species from the 15th to the 30th year, and its coverage, biomasses of aboveground and roots were the highest at the 30^-^ year site (*F* = 7.58, *P*<0.01). To evaluate the performance of soil PSD and D over successional time, the changes of PSD and *D* in the rhizospheres and bulk soils of *A*. *capillaries* and *A*. *sacrorum* were investigated.

**Fig 1:**
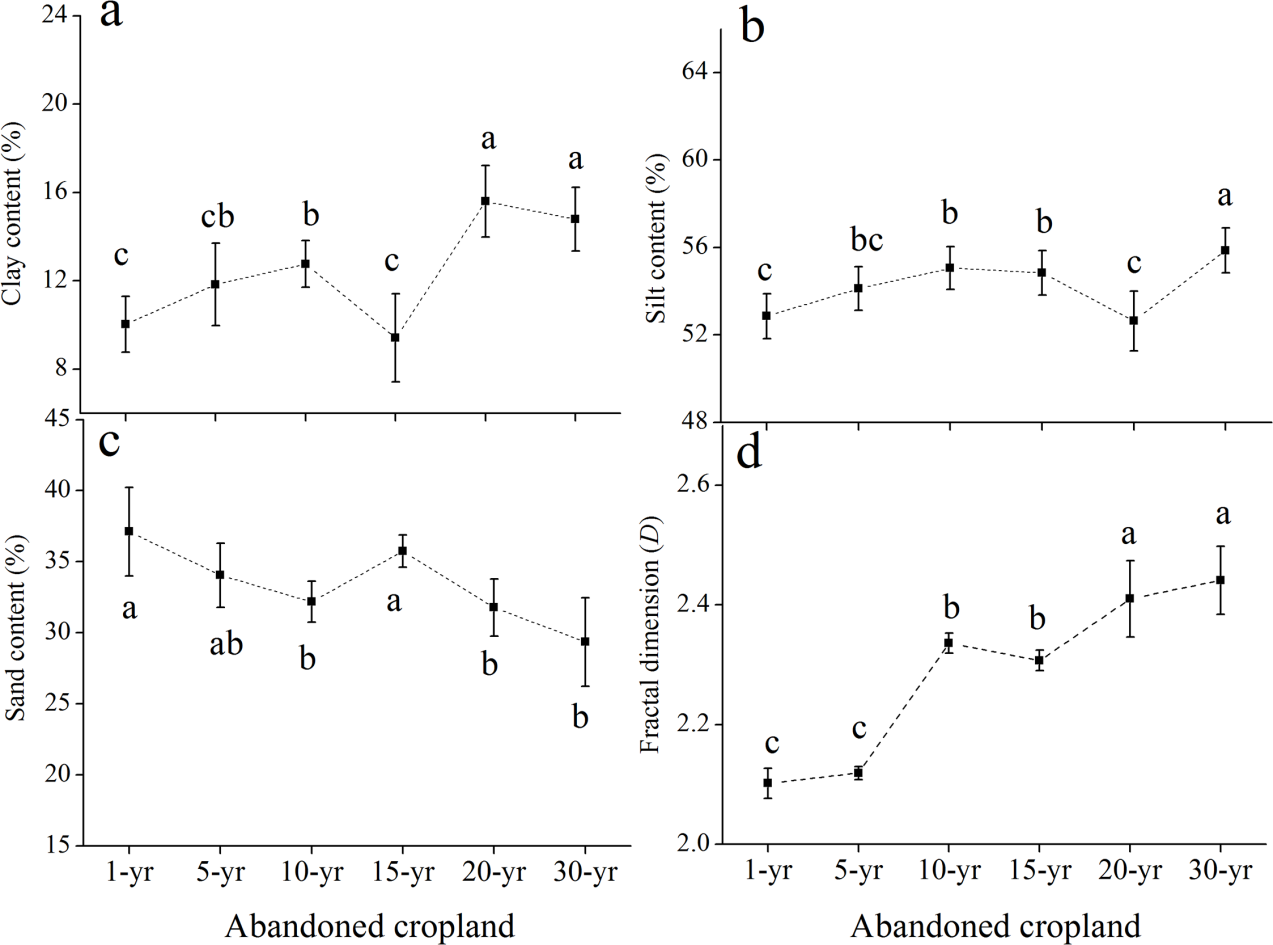
Change of particle-size distribution and fractal dimension in the rhizosphere soils during the natural succession (a-d). Vertical bars indicate standard deviations of means (n = 3). Different letters indicate the significant difference at *p*<0.05.

**Fig 2:**
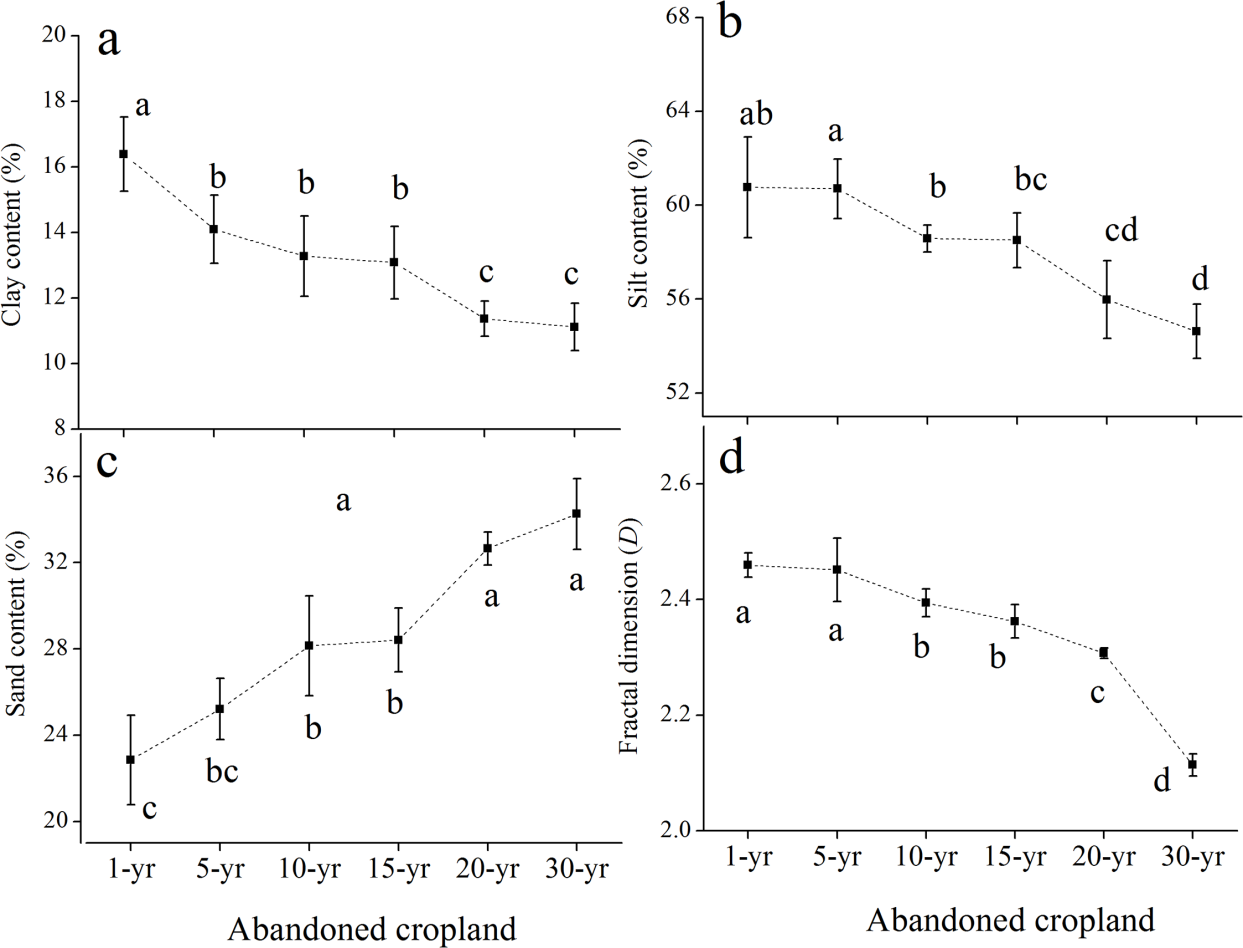
Change of particle-size distribution and fractal dimension in the bulk soils during the natural succession (a-d). Vertical bars indicate standard deviations of means (n = 3). Different letters indicate the significant difference at *p*<0.05.

From Figs 1 and 2. PSD and *D* differed significantly between the two soils at the sites of increasing length of abandonment. In the rhizosphere soil (Fig 1), the clay content significantly increased in the former 10 years and subsequently decreased dramatically at the 15-year site while greatly increased thereafter. Silt content showed the trend of first increasing and then declining in the former 20 years and subsequently increased, reaching the maximum at the 30-yr site (55.87%). In contrast with the clay and silt contents, sand contents in the rhizosphere soil significantly decreased in the former 10 years and considerably increased at the 15-yr site (35.75%), thereafter decreased with time. *D* was generally increased with the successional time. In the bulk soil (Fig 2), the contents of clay and silt presented a decreasing trend with the increasing time while the sand content increased significantly. *D* behaved to the clay and silt contents, declining with time and reaching the minimum at the 30-year site (2.214).

### Relationship between *D* and selected soil properties

**Fig 3:**
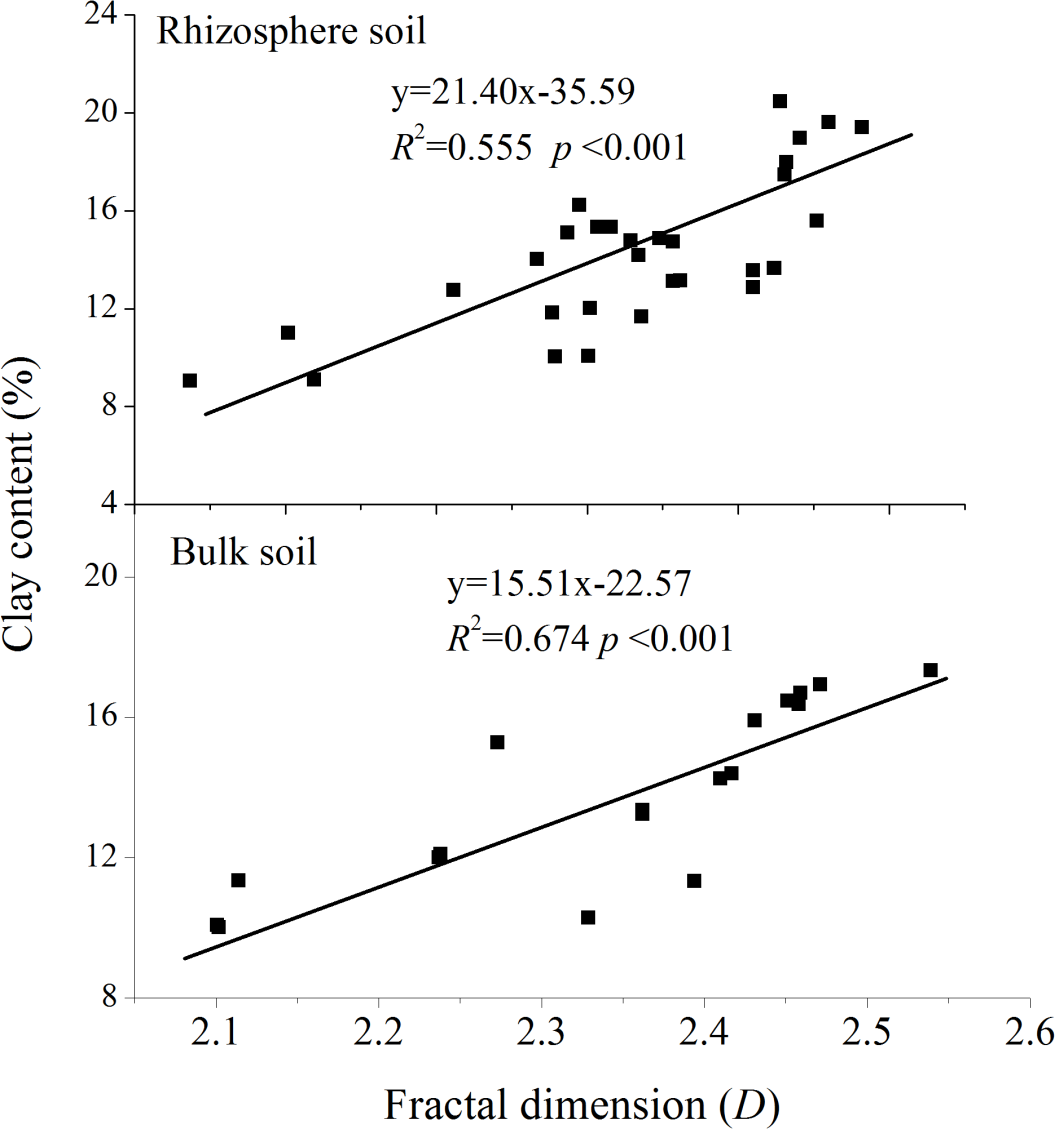
Relationships between fractal dimension and soil clay contents.

**Fig 4:**
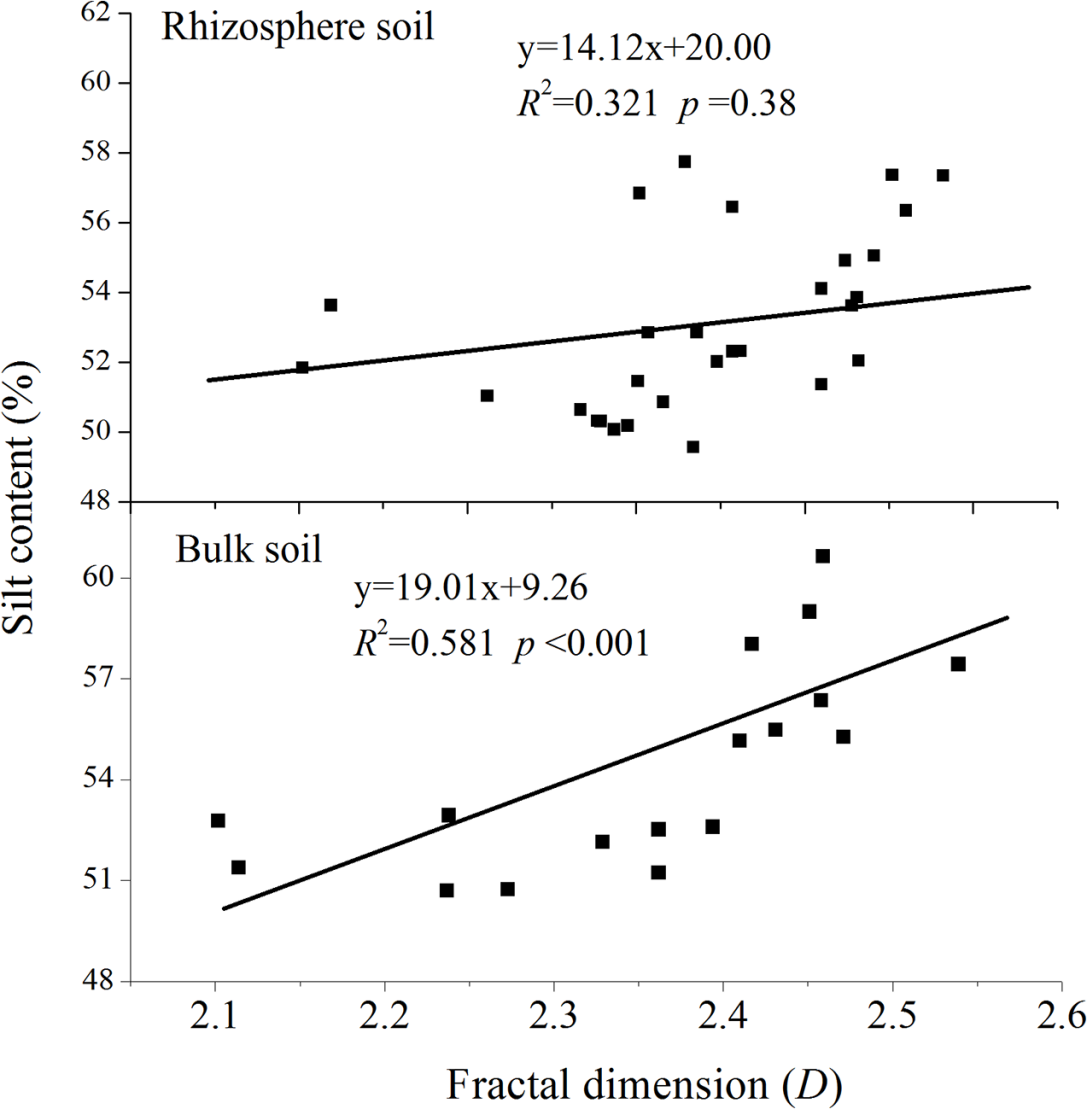
Relationships between fractal dimension and soil silt contents.

**Fig 5:**
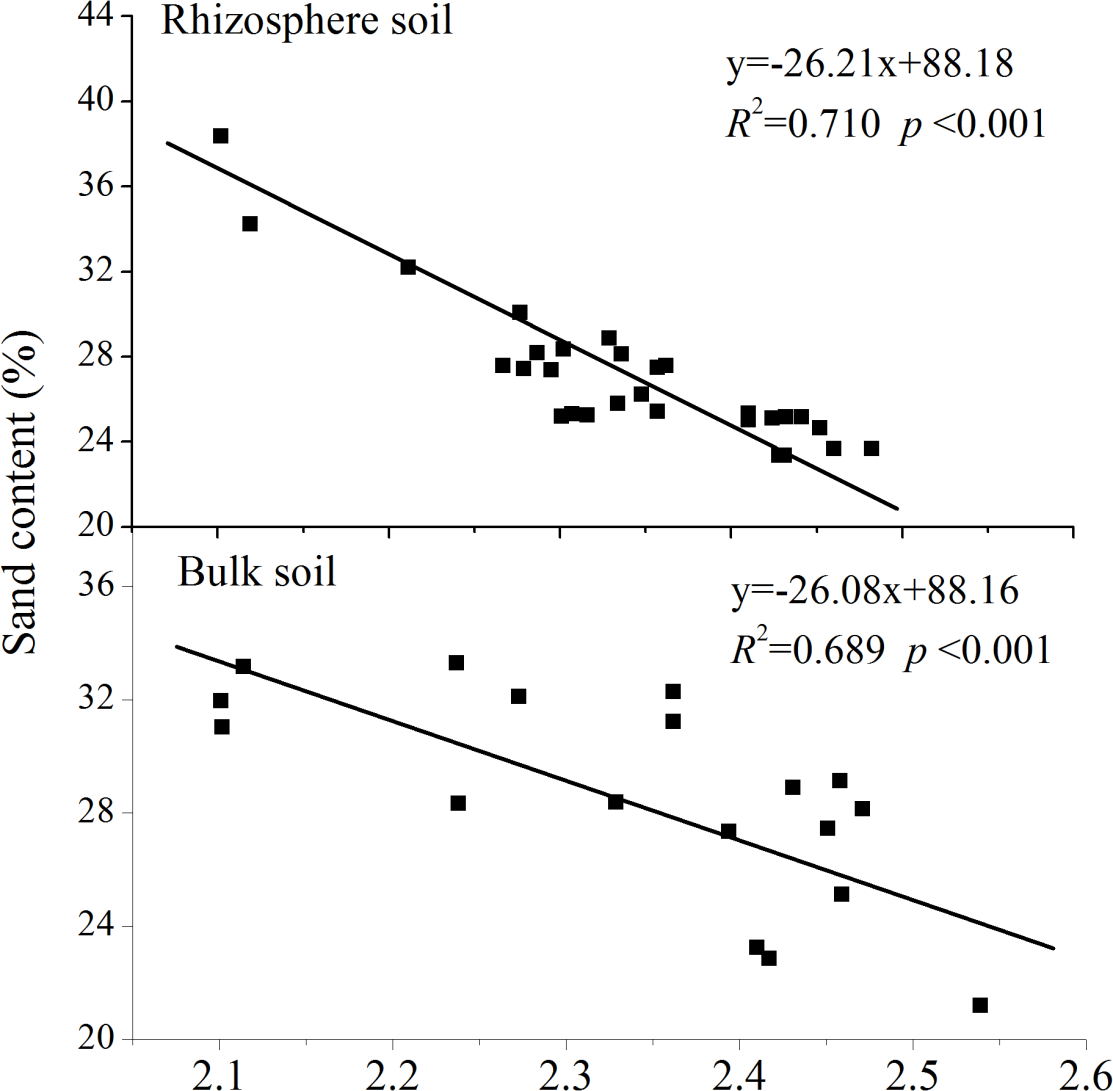
Relationships between fractal dimension and soil sand contents.

**Fig 6:**
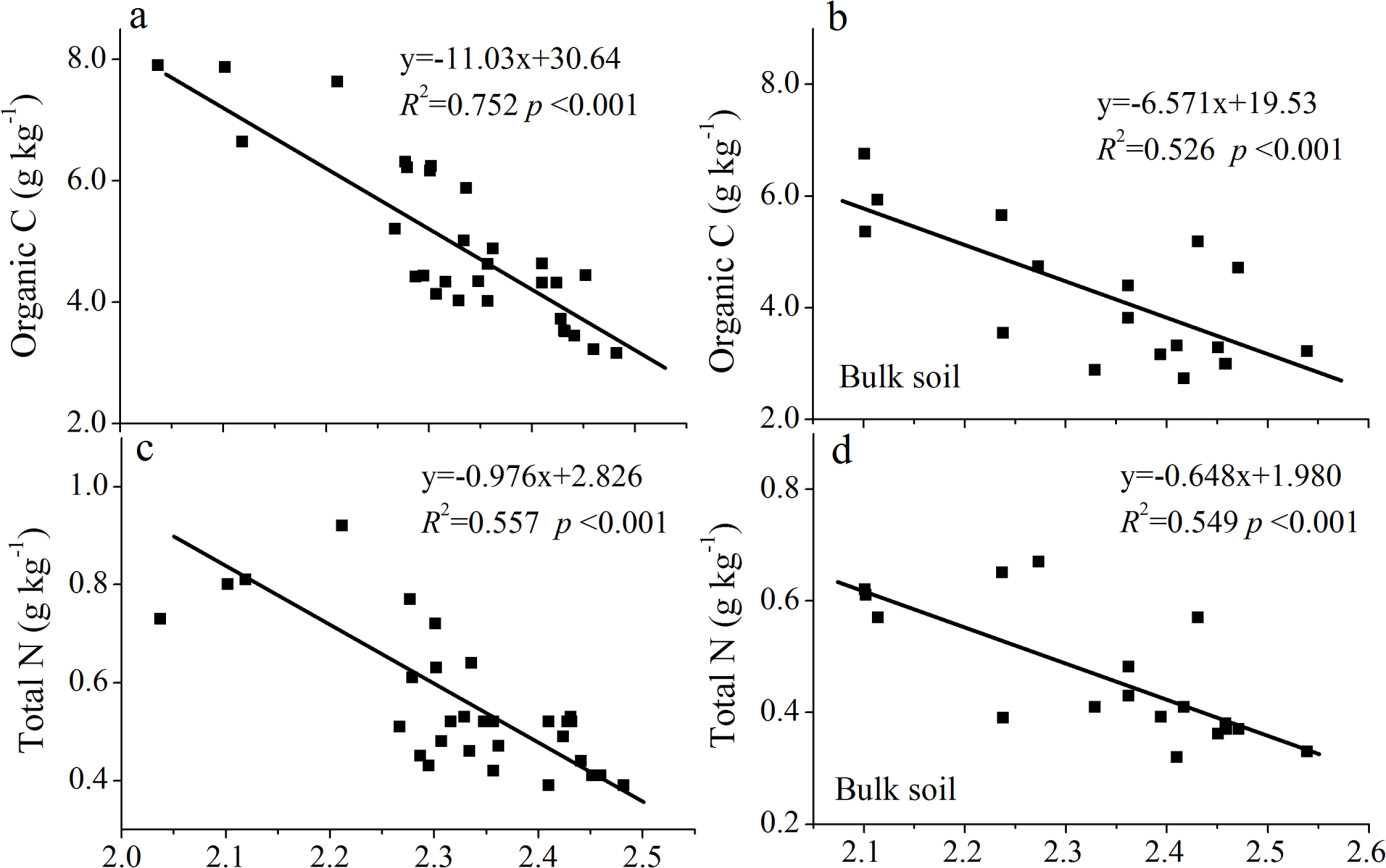
Relationships between fractal dimension, organic C and total N in the rhizosphere and bulk soils (a-d).

Linear regression analyses determined the relationships between *D* and the clay, silt, sand, organic-C, and total-N contents (Figs 3–6). *D* was linearly correlated positively with clay content and negatively with sand content (Figs 3 and 5, *P*<0.001) in both rhizosphere soil and bulk soil. *D* was not correlated with silt content in the rhizosphere soil (Fig 4, *R* ^2^ = 0.321, *P* = 0.38) but was in the bulk soil (*R* ^2^ = 0.581, *P*<0.001). *D* was negatively correlated with organic-C and total-N contents in both the rhizosphere soil and bulk soil (Fig 6, *P*<0.001). The correlations between the soil particles of the rhizosphere and plant characteristics were analyzed to investigate the effect of roots on the PSD of the rhizospheres. Aboveground and root biomasses were clearly negatively correlated with rhizospheric clay and silt contents but were positively correlated with sand content (Table 4, *P*<0.05).

**Table 4:** Pearson correlation between soil PSD and plant characteristics.

Parameters	Clay	Silt	Sand
Coverage	-0.716*	-0750*	0.831**
Aboveground biomass	-0.803**	-0.781**	0.774**
Root biomass	-0.799**	-0.817**	0.805**

*Correlation is significant at the *p*<0.05 level (2-tailed)

**Correlation is significant at the *p*<0.01 level (2-tailed).

## Discussion

### Characteristics of the PSDs of the rhizospheres and bulk soils

The rhizosphere is the zone of higher microbial turnover and activity because it is adjacent to the plant roots. Rhizospheres and bulk soils thus usually differ in their chemical and physical properties [14]. In the present study, the lower silt content and higher sand content in the rhizosphere soils of dominant and companion species than the bulk soils at the 1–15-year sites suggested that rhizospheres at early to intermediate successional stages have larger particles compared to the bulk soil. This result could be due to the effect of the plant roots on the rhizospheres. Plants roots usually exude substrates such as sugars, acids, hormones, mucilage, sloughed root cells, and C allocated to root-associated symbionts [35]. Some viscous substances exuded by roots, such as polysaccharides, phenolic compounds, and polygalacturonic acid, contribute greatly to the cohesion of soil particles [35, 36]. Zhang et al. [37] investigated the fractal features of rhizospheric microaggregates and PSD under six types of vegetation and found that *D* was significantly lower in the rhizospheres than the bulk soils, similar to the results from our study for the 1–15 year sites, indicating that the early to intermediate stages of succession are more seriously affected by the plant roots. As succession proceeded, however, *D* and the clay, silt, and sand contents did not differ significantly between the rhizosphere soil and bulk soil, although the root biomass of the dominant species *(A*. *sacrorum*) was highest (*P*<0.01)at the 30-year site (Table 1). This phenomenon could be related to the accumulation of sand particles and a decrease of silt particles in the bulk soils (Fig 2). Previous studies [38, 39] have shown that soil conditions have greatly improved over the 30 years of restoration on the Loess Plateau, and the increased vegetation coverage has improved the soil physical properties (e.g. aggregate status, sand content, anti-erodibity, and porosity) relative to the early and intermediate stages. Especially at the middle-late stage, more species emerged into the vegetation community and the coverage greatly enhanced. The increased plants roots aggregated smaller soil particles into larger particles by penetration and fixation, which lead to the increase of sand particles and a decrease of silt particles in the bulk soils. Clay content was higher in the rhizosphere than the bulk soil at the 1-year site but did not differ significantly between soils at the 5- and 10-year sites. This is because the dominant pioneering species of the vegetation community at this stage, *A*. *capillaries*, was gradually becoming less dominant, providing significantly lower aboveground and root biomasses (Table 1). The decreased penetration and fixation of roots resulted in the lower small-particle content in the rhizosphere soil. Smaller soil particles are more prone to wind erosion [18]. The loss of particles from wind erosion degrades and desertifies the soil. In our study, the higher sand contents and lower silt and clay contents in the bulk soils as abandonment age increased suggested the coarsening of soil structure during the process of natural recovery. Many smaller soil particles aggregate into larger particles, which reduce the loss of fine particles from soil erosion.

A small variation in *D* means the significant change in the soil PSD. In our work, the *D*s of rhizosphere and bulk soil ranged from 2.102 to 2.441 and from 2.214 to 2.459, respectively, which was consistent with the scaling domain of other studies in China where limits of fragmentation fractal dimension were given as 2<D<3 [11–13]. A decrease of the *D* value in bulk soil indicates a depletion of fine particles and an accumulation of the coarse fraction, which, in the study area, is highly indicative of better soil permeability [7, 40].The clay contents in our study were generally higher in the rhizospheres and lower in the bulk soils as abandonment age increased, except at the 15-year site where the clay and silt were drastically lower and sand content was higher. This discrepancy at the 15-year site may have been associated with the change of the vegetation community. The vegetation during the 30 years of restoration transformed from a community dominated by *A*. *capillaries* to a community dominated by *A*. *sacrorum*. The 15-year site was at the stage at which the dominance changes. The coverage and aboveground and root biomasses of *A*. *sacrorum* were considerably higher at this stage, which likely decreased the *A*. *sacrorum* rhizospheric clay content and drastically increased the sand content. The positive correlation between root biomass and sand content and the negative correlation between root biomass and silt and clay contents support this scenario.

### Characteristics of *D* and its relationship with selected soil properties

In our study, the *D*s of the rhizosphere soil of two dominant species, *A*. *capillaries* and *A*. *sacrorum*, had opposite trends to that of bulk soil. Rhizospheric *D* tended to be higher as abandonment age increased but bulk-soil *D* tended to be lower, perhaps due to the influence of the plant roots. Yang et al. [41] reported that *D* implied the extent of particle coarsening, and a higher *D* usually indicated soils with high compactibility and poor permeability. A higher *D* also implies a higher probability of wind erosion due to the larger proportion of clay particles. The lower *D* of the bulk soils accordingly suggests the accumulation of sand particles, the aggregation of fine particles, and the recovery of soil properties as natural succession progresses, consistent with the findings by Zhou et al. [36] who investigated the fractal characterization of soil microaggregates for grasslands with different restoration times on the Loess Plateau. The higher rhizospheric *D* indicated an accumulation of smaller particles, which was verified by the higher clay and silt contents.

In agreement with previous studies that *D* was highly correlated with clay and sand content in the bulk soils [6, 7], our study found that *D* of bulk soil was linearly correlated positively with clay content and negatively with sand content (*P*<0.001). The rhizospheres had similar relationships. These results indicated that the *D* of soil particles is directly proportional to the clay content and inversely proportional to the sand content. In addition, significant relationships between *D* and organic-C and total-N contents for both two soils suggested that *D* can be used to assess the conditions of PSD and nutrient contents of both rhizospheres and bulk soils.

## Conclusions

PSD and *D* differed significantly between the rhizosphere and bulk soil during natural succession on abandoned cropland on the Loess Plateau due to the influence of the plant roots. The rhizosphere had a higher sand content and a lower silt content and *D* in the early and intermediate successional stages (1–15 years). Clay and silt contents and *D* tended to be higher and sand content tended to be lower in the rhizosphere at longer abandonment ages. The bulk soils had an opposite trend. *D* was linearly correlated with clay, sand, organic-C, and total-N contents for both soils, suggesting that the *D* of PSD was sensitive to the dynamics of PSD and the C and N contents of different soil zones. *D* was a sensitive and practical index for quantifying changes in soil properties. This study has significant implications for the application of the fractal method for describing soil structure and the status of soil nutrients and also provides information for understanding the performance of rhizospheres during ecological restoration.
