# Correction: Fractal Feature of Particle-Size Distribution in the Rhizospheres and Bulk Soils during Natural Recovery on the Loess Plateau, China

**DOI:** 10.1371/journal.pone.0140305

**Published:** 2015-10-05

**Authors:** The PLOS ONE Staff

The Funding section is incorrect. The publisher apologizes for the error. The correct funding information is as follows: This work was financially supported by the National Natural Sciences Foundation of China (41401621), the Science and Technology Research and Development Program of Shaanxi Province (2014JQ5171), and the Fundamental Research Funds for the Central Universities (2452015088).
